# Sex differences in chronic pain-induced mental disorders: Mechanisms of cerebral circuitry

**DOI:** 10.3389/fnmol.2023.1102808

**Published:** 2023-02-20

**Authors:** Zuqi Shen, Wei Li, Weiqi Chang, Na Yue, Jin Yu

**Affiliations:** ^1^Department of Integrative Medicine and Neurobiology, School of Basic Medical Sciences, Shanghai Medical College, Fudan University, Shanghai, China; ^2^Weifang Maternal and Child Health Hospital, Weifang, China; ^3^Shanghai Key Laboratory of Acupuncture Mechanism and Acupoint Function, Fudan University, Shanghai, China

**Keywords:** sex differences, pain, anxiety, depression, neural circuit

## Abstract

Mental disorders such as anxiety and depression induced by chronic pain are common in clinical practice, and there are significant sex differences in their epidemiology. However, the circuit mechanism of this difference has not been fully studied, as preclinical studies have traditionally excluded female rodents. Recently, this oversight has begun to be resolved and studies including male and female rodents are revealing sex differences in the neurobiological processes behind mental disorder features. This paper reviews the structural functions involved in the injury perception circuit and advanced emotional cortex circuit. In addition, we also summarize the latest breakthroughs and insights into sex differences in neuromodulation through endogenous dopamine, 5-hydroxytryptamine, GABAergic inhibition, norepinephrine, and peptide pathways like oxytocin, as well as their receptors. By comparing sex differences, we hope to identify new therapeutic targets to offer safer and more effective treatments.

## 1. Introduction

Chronic pain has a high comorbidity rate with anxiety and depression, and the incidence is as high as 50% (Vos et al., 2020). Epidemiology suggests that there are sex differences in pain-induced mental disorders, and sex factors also affect the efficacy of clinical anti-anxiety and antidepressant drugs (Sramek et al., 2016; LeGates et al., 2019). Most of the experimental studies in neuroscience are carried out in male animals. The excessive dependence on male animals and cells in preclinical studies may mask the key sex differences that may guide clinical research (Madla et al., 2021). Therefore, this paper reviews the research on sex differences in the field of pain and related mental behaviors in recent years, analyzes the sex differences of dopamine, serotonin, GABA, oxytocin, and norepinephrine pathways involved in pain and related emotional behaviors, and combs out the brain circuit mechanism that may be involved in the regulation of sex differences in pain-induced mental disorders. In the era of individualized medical care, emphasis on sex medicine is essential to promote personalized care for patients.

## 2. Sex differences in neural circuits

**Figure 1:**
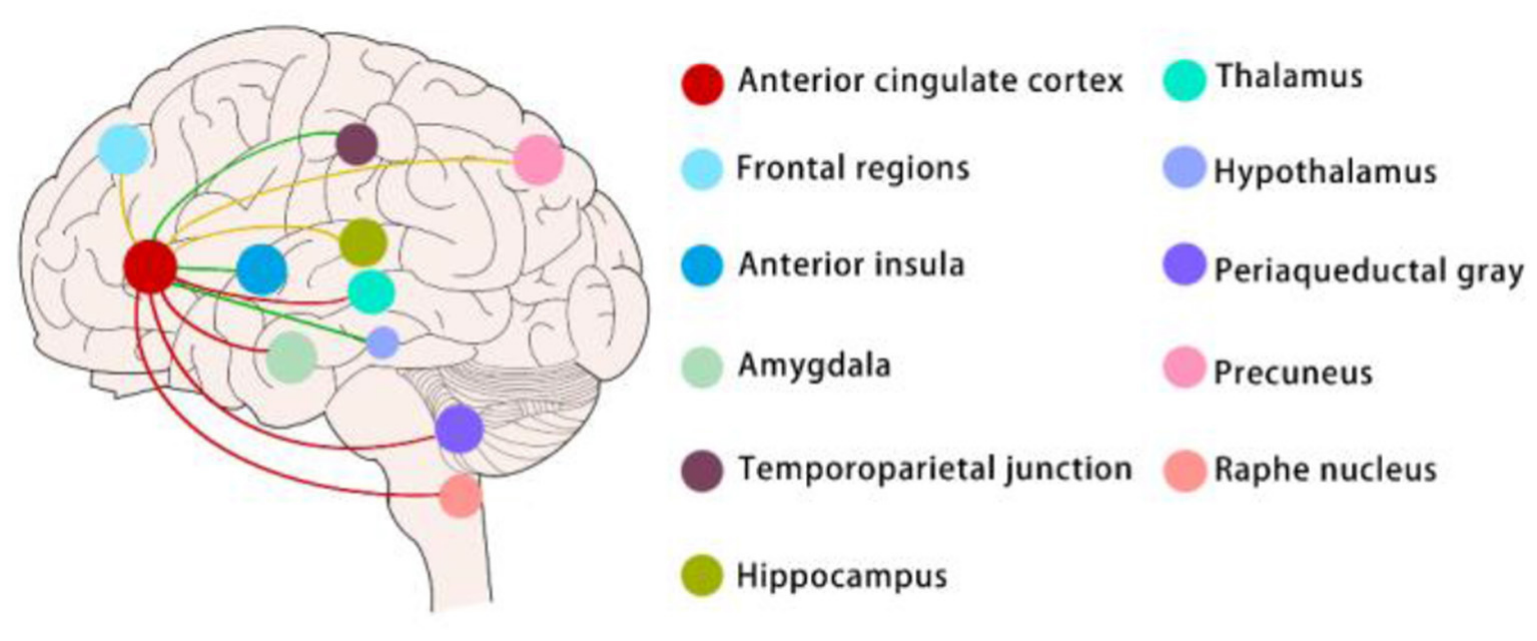
Sex differences in neural circuits (mainly in clinical experiments).

**Figure 2:**
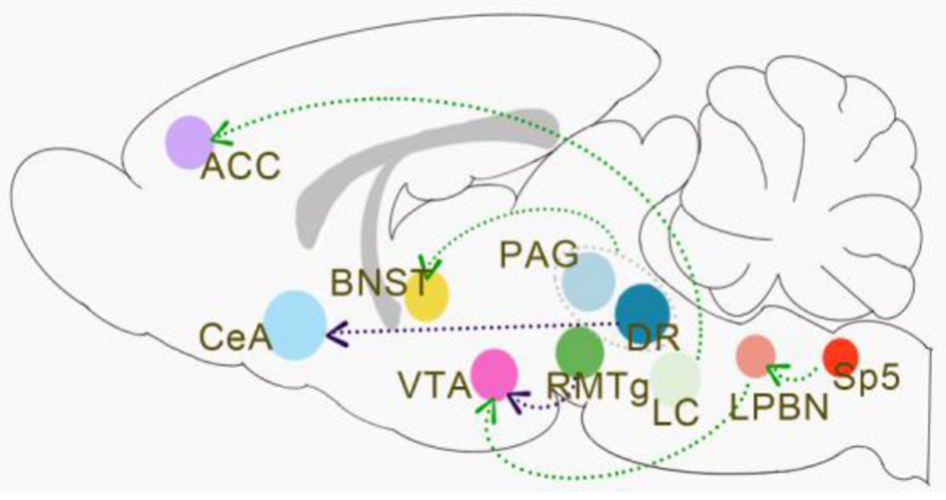
Sex differences in neural circuits (mainly in preclinical experiments).

Many brain regions, such as the anterior cingulate cortex, thalamus, amygdala, medial prefrontal cortex, and periaqueductal gray, are involved in the regulation of both chronic pain and emotions (Bushnell et al., 2013). However, a growing number of researchers believe that no neuron is an isolated island and that the connections between brain regions are more critical than brain subdivisions (Han and Domaille, 2022). Identifying specific or shared circuits that regulate pain and emotions is the key to unraveling the complex manifestations of pain and emotions. In clinical experiments, graph theory, modular analysis, and machine learning were used to analyze the default mode, central, visual, and sensorimotor modules in patients with chronic pain (Figure 1). In preclinical experiments, there were also more nuanced circuit manipulation experiments which were now being carried out extensively in animal experiments (Figure 2). Interestingly, both the clinical and preclinical results showed that sex difference was a non-negligible factor in the study of pain and emotions (Mogil, 2020). Brain regions and connections related to pain and emotions such as anterior cingulate cortex, amygdala, locus coeruleus, ventral tegmental area, and periaqueductal gray were all sexually differentiated.

Sex-difference neural circuits mentioned in this article are mainly about the functional connectivity (FC) of ACC. The red lines indicate that women exhibit greater FC between the anterior cingulate cortex and the thalamus, amygdala, periaqueductal gray, and raphe nucleus than men when chronic pain and pain emotions exist; The green lines indicate that men exhibit greater FC between the anterior cingulate cortex and temporoparietal junction, anterior insula, and hypothalamus than women when chronic pain and pain emotions exist. The yellow lines indicate that only women with chronic pain had greater ACC FC to the precuneus and lower FC to the hippocampus and frontal regions, men with chronic pain have no change in these connectivities compared to healthy men.

The green lines indicate that activation of the pathways induces pain-related depression in males but not females. Activation of the LC → ACC pathway leads to pain-induced depression in males, but it remains unknown whether the LC → ACC pathway conducts the same function in females. Increased VTA DA neuronal activity was associated with chronic neuropathic pain-induced depression-like behaviors. The activation of the Sp5C-LPBN^Glu^-VTA^DA^ pathway was directly involved in the modulation of pain-related depression in males, but this circuit was not manipulated in females. Activation of the terminals in vlPAG/DR^DA+^ neurons or vlPAG/DR^DA+^-BNST can reduce nociceptive sensitivity in naïve male mice and inflammatory pain states in males, whereas in female mice resulted in increased locomotion in the presence of significant stimuli. The purple lines indicate that activation of the pathways induces pain-related depression both in males and females. The inhibition of DRN serotonergic neurons → CeA somatostatin neurons pathway produces depression-like behaviors both in male and female mice models with chronic pain. There are also no sex differences in the regulation of the RMTg^GABA^-VTA^DA^ circuit for chronic pain-induced pleasure deficits.

### 2.1. Anterior cingulate cortex (ACC)

Recent studies indicate that the anterior cingulate cortex (ACC) plays a critical role in chronic pain and pain-related emotional responses (Li et al., 2021). As an important part of the limbic system, ACC involves in pain and pain-related emotions *via* connectivity with other brain regions such as the descending pain antinociceptive system and so on (Chen et al., 2021). In a cross-sectional resting-state functional connectivity (RSFC) study among older adults, researchers found that the strongest evidence for sex differences emerged in the associations of thermal pain with RSFC between the ACC and amygdala and between the ACC and PAG in older females relative to older males (Figure 1) (Monroe et al., 2018). Researchers also investigated whether women have stronger functional connectivity (FC) and greater structural connectivity (SC) compared to men between the subgenual ACC (sgACC) and the descending antinociceptive system. They revealed that brain circuitry in women may provide for greater engagement of the descending modulation system mediating pain habituation but not in men. Between the sgACC and the periaqueductal gray (PAG), raphe nucleus, medial thalamus, and anterior midcingulate cortex (aMCC) women exhibited greater FC than men (Figure 1) (Wang et al., 2014). There were also findings indicating that abnormal sgACC circuitry is unique to women but not men with ankylosing spondylitis-related chronic pain. Compared to men, women had greater sgACC FC to the default mode and sensorimotor networks (Figure 1) (Osborne et al., 2021). Besides the connectivity, the anatomy of the ACC of rats was also sexually dimorphic with males having greater dendritic spine density as well as arborization. And this reduction was more pronounced for males with increasing age (Markham and Juraska, 2002). Synaptic plasticity is a key cellular mechanism for pain perception and emotional regulation. In preclinical studies, excitatory transmission and plasticity in the anterior cingulate cortex are critical in chronic pain-related emotions. Researchers used a 64-channel multielectrode (MED64) system to record synaptic plasticity in the ACC and found that long-term depression (LTD) was greater in ACC in male mice than females while long-term potentiation (LTP) did not show a sex-related difference (Liu et al., 2020). Besides, some studies reported that Sex differences in GABAergic gene expression occur in the ACC in schizophrenia (Bristow et al., 2015). It remains unknown whether Sex-difference GABAergic gene expression involves in pain and pain-related emotion.

### 2.2. Amygdala

It has been suggested that the amygdala receives inputs from the parabrachial nucleus and mediates differentiated pain and affective responses in males and females (Sun et al., 2020). Calcitonin gene-related peptide 1 (CGRP1) receptors in the central nucleus of the amygdala (CeA) are involved in neuropathic pain-related amygdala activity and contribute to nociception in both sexes (Presto and Neugebauer, 2022). However, they elicit emotional-affective pain responses such as ultrasound onset and anxiety-like behaviors mainly in females (Neugebauer et al., 2020). The dorsal raphe nucleus (DRN) is the main brain region for the synthesis and release of serotonin, and its involvement in pain-affective reactions has been discussed previously. It has been suggested that the inhibition of DRN serotonergic neurons → CeA somatostatin neurons pathway produces depression-like behaviors in male mice models with chronic pain. Activation of this pathway using pharmacological or optogenetic approaches reduced depression-like behavior in these mice (Figure 2) (Zhou et al., 2019). In this study, the researchers also included MRI data of the patient's FC to corroborate this finding. However, these data were not sex-differentiated, which seems to indicate that this circuit is not sexually dimorphic.

### 2.3. Locus coeruleus (LC)

The locus coeruleus (LC) acts as a nucleus that regulates pain and emotion (Hirschberg et al., 2017). Resilience to chronic stress is mediated by the noradrenergic regulation of dopamine neurons (Llorca-Torralba et al., 2016). The circuits in which it is involved have also received considerable attention. In studies performed on male rats only, it was believed that bilateral chemogenetic inhibition of the LC → ACC pathway relieves pain-induced depression (Figure 2) (Llorca-Torralba et al., 2022), while activation of the noradrenergic LC → spinal cord pathway relieves pain. However, the noradrenergic system is considered to be sexually dimorphic in much of the literature. Studies have shown differences in the structure and function of LC between male and female rodents in many ways (Bangasser et al., 2015). The dendrites of the neurons in the LC of female rodents were more complex than those of the males (Bangasser et al., 2011), and the female LC dendrites further extended to the areas around the LC, as well as the afferent limbic systems involved in the stress response, such as the central nucleus of the amygdala and the bed nucleus of the stria terminalis (Van Bockstaele et al., 2001). And due to the increased synaptic density in females relative to males, female LC dendrites may receive more synaptic input (Bangasser et al., 2011). This means that anatomically the noradrenergic neurons of the LC may then form different circuits from other brain regions. Therefore, it remains unknown whether inhibition of the LC → ACC pathway relieves pain-induced depression is applicable to female rats.

### 2.4. Ventral tegmental area (VTA)

In addition to the involvement of the LC noradrenergic system in pain and depression co-morbidity, dopaminergic neuronal projections from the ventral tegmental area (VTA) to the prefrontal cortex (PFC), amygdala and nucleus ambiguus (NAc) play a key role in the perception and regulation of chronic pain symptoms (Hipólito et al., 2015). Rostromedial tegmental nucleus (RMTg) GABA hyperinhibition of the VTA dopamine (DA) neurons mediates pain-induced pleasure deprivation (Figure 2). In their experiments, the researchers concluded that no differences were found in the behaviors of males and females in the disease state or the alterations in their behaviors after the manipulation of the circuits (Markovic et al., 2021). This suggests that there are no sex differences in the regulation of the RMTg GABA-VTA DA circuit for chronic pain-induced pleasure deficits (Figure 2) (Lowes et al., 2021). In another study, it was suggested that chronic neuropathic pain-induced depression-like behaviors were associated with increased VTA DA neuronal activity and that upstream spinal trigeminal sub-nucleus caudalis to the lateral parabrachial nucleus (Sp5C-LPBN) glutamatergic neuronal projections were directly involved in the modulation of VTA DA neurons (Figure 2) (Zhang et al., 2021). However, this circuit was not manipulated in female rats. Some studies support that the projections, gene expression levels, and electrical activity of VTA DA neurons do not differ significantly in males and females, which may explain the absence of functional differences in VTA DA-involved circuits in the co-morbidity of chronic pain and depression (Chung et al., 2017).

### 2.5. Periaqueductal gray (PAG)

In contrast, the DA neuron to the bed nucleus of the stria terminalis (BNST) projection circuit in the ventral lateral aqueduct periaqueductal gray/dorsal fissure (vlPAG/DR) has male and female differences in pain-related behaviors (Figure 2) (Yu et al., 2021). It has been found that activation of the terminals in vlPAG/DR^DA+^ neurons or vlPAG/DR^DA+^-BNST can reduce nociceptive sensitivity in naïve male mice and inflammatory pain states in males, whereas activation of this pathway in female mice resulted in increased locomotion in the presence of significant stimuli (Yu et al., 2021). There is still insufficient fundamental research to answer the question of whether DA in PAG/DR is indeed sex-differentiated in projections and functions. Early studies suggested that the projection from the PAG to the rostral ventromedial medulla (RVM) is sexually dimorphic and that systemic administration of morphine significantly inhibited pain-induced PAG activation in male rather than female rats. It has also been suggested that microglia (Doyle et al., 2017) in the PAG, opioid receptor signaling (Loyd and Murphy, 2006), morphine metabolites (Doyle and Murphy, 2018), and endocannabinoids (Llorente-Berzal et al., 2022) are all sexually differentiated in their involvement in pain regulation. The PAG is a key structure in a number of regulatory pathways of nociception, emitting neuro fibers projections to the amygdala, hypothalamus, frontal cortex, hippocampus, and BNST to regulate the generation of pain and pain-related behaviors. We thus hypothesize, with little knowledge, that the brain circuits involving PAG are more likely to exhibit sexual dimorphism in their involvement in pain and related emotions.

In conclusion, whether different brain circuits are sexually dimorphic in their involvement in pain and related emotions may be related to differences in their own anatomies, molecular levels, and electrophysiological activities *per se*, and cannot be generalized in a simple way.

## 3. Sex differences in neurotransmitters and neuromodulators

The past and current literature have indicated that neurotransmitters and neuromodulators systems such as norepinephrine (Joshi and Chandler, 2020), dopamine (Hasbi et al., 2020), serotonin (Zhang et al., 2017), GABA (Cerne et al., 2022), and oxytocin (Tamborski et al., 2016; Aulino and Caldwell, 2020) seemed to be strongly involved in pain-related sexually dimorphic mental disorders (Dazzi and Scicchitano, 2014). Differences in concentrations (Busch et al., 1997), receptors (Hasbi et al., 2020), and transporters (Zachry et al., 2021) of these neurotransmitters and neuromodulators sexual differences may be potential targets for explaining sex differences in pain-related mental disorders.

### 3.1. Norepinephrine

Norepinephrine (NE) mediates the pathogenesis of pain and anxiety co-morbidity (Phillips et al., 2018). Serotonin-noradrenaline reuptake inhibitors (SNRI) antidepressants are widely used in anxiety disorders and they block the reuptake of NE and 5-HT, making them psychotropic drugs for the treatment of neuropathic pain in clinical settings as well (Fava et al., 2018). Norepinephrinergic neurons are mainly found in the LC and widely project to the cerebral cortex, hippocampus, hypothalamus, cerebellum, brainstem nuclei, and spinal cord (Mason, 1979). Previous studies have suggested that sex differences in the locus coeruleus noradrenergic system (Bangasser et al., 2015; Mulvey et al., 2018; Joshi and Chandler, 2020) may be one of the reasons why pain and depression occur frequently in females.

It has been reported that the LC of adult female rats is larger than that of male rats (Pinos et al., 2001) and contains more NE-ergic neurons (Guillamón et al., 1988), which corresponds to the phenomenon in humans (Busch et al., 1997). This may have increased the capacity for NE generation and release in females. In addition to differences in the number of neurons, there are also sex differences in the LC dendritic morphology (Bangasser et al., 2011). Morphological analysis of individual LC neurons by the researchers revealed that female LC dendrites are longer and more complex than those of males, which may increase synaptic afferent contacts in the peri-LC region (Mulvey et al., 2018). For example, increased nociception introduced by PAG (Bangasser et al., 2011) may be one of the neurobiological mechanisms underlying the sex differences in pain.

Apart from the differences in the number and morphology of NE-ergic neurons in LC, it has been reported that estradiol treatment increases NE levels in the ventral hippocampus, cortex, and hypothalamus of ovariectomized female rats (Bangasser et al., 2011). In addition, estrogen can increase NE synthesis and decrease NE degradation, while ovarian hormones increase NE levels in LC target regions through presynaptic modulation of NE release (Vathy and Etgen, 1988). This can occur through estrogenic regulation of the NE biosynthetic enzyme tyrosine hydroxylase (TH) (Serova et al., 2002; Dalla et al., 2010). The widespread projection system of the LC can release NE into the forebrain and regulate emotional behaviors by targeting forebrain regions. LC activation in animal models of chronic pain exhibited an anxiolytic-depressive phenotype (Landau et al., 2015) but these experiments did not involve females (Alba-Delgado et al., 2013).

However, in reports on other excitatory mediators associated with LC and neuropathic pain, the main excitatory neurotransmitter of the associated stress response, corticotropin-releasing factor (CRF), was enhanced in LC with sex differences. The expression of the CRF1 receptor was increased in the LC of male mice (Bangasser et al., 2013) with chronic pain and anxiogenic phenotypes, whereas this receptor was weakly expressed in anxiety-resilient female mice with pain. Interestingly, increased sensitivity of LC to CRF signaling in females leads to enhanced LC responses of females to non-pain stresses.

In conclusion, sex differences in the locus coeruleus norepinephrine system in the phenotype of pain and related anxiety and depression is an area of research interest.

### 3.2. Dopamine

Dopaminergic neurons in the brain are widely distributed in the substantia nigra pars compacta (Poulin et al., 2018), ventral tegmental area (Markovic et al., 2021), hypothalamus (Kim et al., 2019) and periventricular area, periaqueductal gray, dorsal raphe nucleus (Yu et al., 2021), and the olfactory bulb (Pignatelli and Belluzzi, 2017). Regions such as the prefrontal cortex (Bhattacherjee et al., 2019), striatum (Dentresangle et al., 2001), nucleus ambiguus, amygdala (Janak and Tye, 2015), thalamus, hippocampus, periaqueductal gray (Yu et al., 2021), and dorsal horn of the spinal cord are all innervated by dopaminergic neurons and are involved in the transduction of pain and related behaviors (Mercer Lindsay et al., 2021; Yang H. et al., 2021). The nigrostriatal dopaminergic system affects pain transduction and perception through the ascending and descending pathways (Dieb et al., 2016). The mesolimbic dopaminergic system regulates pain perception mainly through the reward or motivational pathways in the ventral tegmental area innervating the vomeronasal nucleus, amygdala, thalamus, and hippocampus (Serafini et al., 2020). It also influences learning and memory as well as sensory evaluation of pain through projections to the prefrontal cortex (Huang et al., 2020).

In recent years, it has become a consensus that there are sex differences in the incidence of dopamine-related neuropsychiatric disorders and sensitivity to dopamine-enhancing drugs such as stimulants, and previous studies have shown that dopaminergic circuits often act through dopamine receptors D1 and D2 (Fasano et al., 2013; Stalter et al., 2020; Allichon et al., 2021). It has been shown in the chronic constriction injury (CCI) pain model of the sciatic nerve in mice that dopaminergic projections from the VTA to the nucleus accumbens (NAc) are involved in pain modulation (Ding et al., 2021). In addition, optogenetic activation of dopaminergic neurons in the VTA and their nerve endings in the NAc significantly increases the nociceptive threshold in CCI rats, and the analgesic effect is exerted mainly through D2 (Gao et al., 2020). D1 and D2 may be involved in pain and analgesia in different ways. In the PAG, an important component of the nociceptive descending regulatory system, the analgesic efficacy of opioid receptor agonists on thermal pain stimuli in mice is significantly reduced following damage to dopaminergic nerve endings. Microinjection of D1 antagonists attenuated the analgesic effect of opioids in the hot plate tests, whereas D2 antagonists showed no such effect (Tobaldini et al., 2018). In another study, pharmacological experiments revealed that both D1 and D2 antagonists significantly antagonized the analgesic effects of opioids. Injections of D2 agonists into PAG increased the nociceptive threshold in mice, and the analgesic effects of D2 agonists were blocked by D2 antagonists and γ-aminobutyric acid receptor agonists or opioid receptor antagonists. This study also demonstrated that the combination of D1 and D2 agonists had a greater anti-injurious effect than any one of the receptor agonists alone (Wang et al., 2021).

More interestingly, a number of studies have found that D1 and D2 are involved in sex-differentiated regulation of pain and related behaviors such as anxiety and depression (Hasbi et al., 2020). For example, in the caudate nucleus of non-human primates and the rat striatum, females express a higher density of D1–D2 heteromeric complexes and more D1–D2-expressing neurons compared to males (Hasbi et al., 2020). Signaling pathway analysis showed that sex differences in D1–D2 heteromer expression resulted in differences in the basal and heteromer-stimulated activity of two important signaling pathways, BDNF/TrkB and Akt/GSK3/β-linked protein. The dopamine D1–D2 heteromeric complex is involved in depressive and anxiety-like behaviors, and higher D1–D2 heteromer expression in females may significantly increase the propensity for depressive- and anxiety-like behaviors (Hasbi et al., 2020). Furthermore, in the field of non-pain-related emotions, it has been demonstrated that sex differences in D1 receptor-regulated molecular pathways lead to sex differences in social withdrawal behaviors (Campi et al., 2014). Although social defeat increased dopamine levels in male and female NAc, social withdrawal was induced only in female California mice, but not in male ones. Pharmacological experiments showed that D1 receptor activation was sufficient to induce social withdrawal in females, but not in males. D1 antagonists increased social approach behavior in females exposed to social defeat but did not affect naïve females (Campi et al., 2014). Apart from the fact that D1 and D2 are differentially expressed between males and females, they may be a potential target for revealing the differences in pain-related emotions between the two sexes. Sex differences in neurological dopamine sensitivity as well as in the balanced dopamine release among the circuits could be another area of concern.

In clinical and preclinical studies, using pharmacological methods combined with PET-CT imaging, researchers have suggested that the increased sensitivity of the striatal dopamine reward system in females compared to males may underlie the sex differences in substance use disorders and attention-deficit/hyperactivity disorder (ADHD) (Manza et al., 2022). However, some researchers pointed out that there is little sex difference in the encoding of VTA neurons as well as dopamine release and vesicle depletion in the NAc during the learning process of cue-action-reward instrumental tasks, and that dopamine-related sex differences may be mediated by secondary mechanisms that flexibly affect dopamine cell and circuit functions (Rivera-Garcia et al., 2020). Much of the research on the dopamine system has focused on addiction-related disorders, and it has been suggested that sex differences between different dopamine projections underlie sex differences in addiction (Becker, 2016). In rodents, ovariectomized female rats exhibit smaller initial dopamine increases after cocaine treatment than castrated male rats. Estradiol treatment of ovariectomized female rats enhanced stimulated dopamine release in the dorsolateral striatum but not in the vomeronasal nucleus, resulting in sex differences in the balance between these two dopaminergic projections (Becker, 2016). Moreover, it is not clear whether sex differences regarding the balance of the dopaminergic nervous systems are involved in the generation of sex differences in pain and pain-related emotions. It may be an important potential target.

While many of the previous studies have focused on sex differences in the anatomical structure of dopamine neurons and sex differences related to dopamine levels, it has been suggested that how sex differences in microcircuit regulatory mechanisms mediate sex-differentiated dopamine dynamics is worthy of equal attention (Zachry et al., 2021). Studies suggested that there are local regulatory mechanisms in the ventral tegmental area of the midbrain limbic dopamine system to the striatal circuits that are independent of somatic activity and that these processes can occur through both homogeneous synaptic mechanisms (e.g., presynaptic dopamine self-receptors and dopamine transporter proteins) and heterosynaptic mechanisms (e.g., retrograde signaling of postsynaptic cholinergic and GABAergic systems, etc.) (Zachry et al., 2021), so that the dopamine released by the striatal axonal terminals can be independently and rapidly regulated. In addition, these regulations are potential targets of sex differences in ovarian hormone-dependent and non-dependent dopamine regulation (Zachry et al., 2021). These mechanisms have been shown to be key mediators of multiple psychiatric disorders and involved in the expression of sex-specific behaviors.

### 3.3. Serotonin

As a monoamine neurotransmitter, serotonin plays a vital role in regulating emotions (Kraus et al., 2017), learning (Grossman et al., 2022), memory (Wu et al., 2021), sleep (Monti, 2011), and appetite (Blundell, 1984), and is closely related to pain and neuropsychiatric disorders such as major depression and anxiety (Zhou et al., 2022). In recent decades, selective serotonin reuptake inhibitor (SSRI) drugs have been the most commonly prescribed medications for depression. Clinical and preclinical studies suggest that amitriptyline, a tricyclic antidepressant used to treat mood disorders, neuropathic pain, and migraine, can increase serotonin levels and restore behavioral responses associated with pain and depression (Zhang et al., 2017). Long-term administration of fluoxetine, an SSRI, was found to prevent anxiety and depression caused by sciatic nerve injury without affecting mechanical allodynia (Barthas et al., 2017). Researchers had found, but controversially, that SSRIs were more effective for females compared to tricyclic antidepressants (Kornstein et al., 2000). Sex differences regarding the serotonergic system had been much reported. In particular, 5-Hydroxy indoleacetic acid (5-HIAA) was reported to be increased in the cerebrospinal fluid of women suffering from depression (Rubinow et al., 1998). Studies have shown increases in 5-HT activity, 5-HT synthesis, and 5-HT metabolites in the brains of female rats compared to male ones (Carlsson and Carlsson, 1988; Haleem et al., 1990). In a clinical study comparing SSRIs with tricyclics, researchers found that menopause significantly affected treatment outcomes and pointed out that this sex-specific difference may be related to the hormonal milieu (Yonkers and Simoni, 2018). Studies conducted with multiple models of depression such as the Chronic Mild Stress Model, the Learned Helplessness Model, the Flinders Sensitive Line rats, and the Lipopolysaccharide-Induced Sickness Behavior in mice have shown that serotonergic neurochemical responses are affected differently in males and females, resulting in sex-dependent behavioral effects (Serova et al., 2002; Dalla et al., 2010).

In previous studies, researchers have noted that sex steroids such as testosterone, progesterone, estrogen, and HPA axis, all have effects on the serotonin pathway (Songtachalert et al., 2018). Activated immune inflammation induces the indoleamine-2,3-dioxygenase (IDO) and tryptophan catabolite (TRYCAT) pathways, thereby enhancing tryptophan degradation and increasing the generation of TRYCATs, including kynurenine and quinolinic acid, exerting an overall anxiogenic effect. The effect of immune activation on IDO is greater in females than in males, therefore, females are more likely to exhibit elevated anxiogenic TRYCAT levels following immune challenge. Moreover, aberrations in the IDO-activated TRYCAT pathway are observed in pregnant females and parturients and are associated with increased levels of postpartum anxiety (Songtachalert et al., 2018).

In addition to the metabolic pathways of serotonin, studies have also found that there are sex differences in serotonin receptors (Zhang et al., 1999; Snoeren et al., 2014; Yamada et al., 2015). There were also differences in 5-HT_1A_ receptor responses between males and females in the repeated stress restraint model in rats. Only males exhibited elevated 5-HT_1A_ receptor G protein coupling responses after repetitive restraint, whereas only females showed increased 5-HT_1A_ receptor responses in the hippocampus following single or repeated exposure (Philippe et al., 2022). In studies exploring sex-related differences in genetics, stress, and the nervous system, female 5-HT_1B_ receptor knockout mice showed significantly lower immobility time and significantly higher baseline hippocampal 5-HT levels than male 5-HT_1B_ receptor knockout mice or male and female wild-type mice in tail suspension and forced swimming tests (Jones and Lucki, 2005). This suggests that female 5-HT_1B_ receptor knockout mice exhibit sex-related disinhibition of 5-HT release, which maintains higher baseline levels of hippocampal 5-HT and behavioral vulnerability to 5-HT depletion (Jones and Lucki, 2005). Moreover, the serotonin transporter protein is also worthy of investigation as a target for many anxiolytic and antidepressant drugs. Using 5-HT transporter (5-HTT) gene-deficient mice as an anxiety animal model, researchers examined cerebral blood flow during resting and amygdala hyperresponsiveness periods using resting-state functional magnetic resonance imaging (rs-fMRI) (Kolter et al., 2021). The results indicated that amygdala reactivity in 5-HTT-deficient mice is regulated by the 5-HTT genotype in males. Whereas, in females it is regulated by the estrous cycle and the predominant influence of gonadotropins may mask genotypic effects (Kolter et al., 2021).

In conclusion, the role of the serotonin system in the sex-differentiated modulation of pain, anxiety, and depression is a matter worthy of investigation.

### 3.4. Gamma-aminobutyric acid (GABA)

GABA, as one of the important inhibitory neurotransmitters, regulates the encoding of pain and anxiety-depressive mood (Cerne et al., 2022). It has been reported that the anxiolytic effect of GABA depends mainly on its binding to the GABA_A_ receptors (GABA_AR_). The benzodiazepine anxiolytic GABA_AR_ modulators have been in clinical use for decades (Sollozo-Dupont et al., 2015). GABA_AR_ functions through its subunit composition, the activation of which allows GABA to exert trophic effects in immature neurons.

Recent studies found that GABA-mediated responses were sexually dimorphic even in the absence of gonadal hormone and that there were sex differences in the expression of GABA_AR_ subtypes (Mir et al., 2020). Researchers assessed sex differences in GABA_AR_ function of hypothalamic neurons before brain masculinization by gonadal hormones by culturing 16-day rat embryonic ventral medial hypothalamus neurons *in vitro*, combined with calcium imaging and electrophysiological recordings (Mir et al., 2020). Optogenetic-specific activation of the dmPFC/vlPAG neural pathway had been reported to produce analgesic and anxiolytic effects in chronic pain-anxiety mice. dmPFC-specific activation of inhibitory neurons in dmPFC was reported to induce nociception and anxiety under normal conditions and chronic pain, and the GABA_AR_ or mGluR1 antagonists can produce analgesic and anxiolytic effects (Yin et al., 2020). However, this study focused only on male mice and it is unknown whether female ones have the same phenotype.

In studies of disorders associated with altered mPFC functions such as schizophrenia, ADHD (Aoki et al., 2013), post-traumatic stress disorder (Lou et al., 2020), depression (Yang L. et al., 2021), and drug addiction (Jasinska et al., 2015), etc., researchers suggested that sex-differentiated manifestations can be partially explained by sex differences in G protein gated inwardly-rectifying K^+^ (GIRK)-dependent signaling in mPFC pyramidal neurons. Neuronal GIRK channels are formed by homo- or heteromeric assembly of GIRK1/GIRK2/GIRK3 subunits. They play a key role in regulating excitability throughout the brain and are associated with a variety of neurological disorders as well as sex differences in cellular functions. Sex differences in GABA_BR_-GIRK signaling are attributed to a phosphorylation-dependent transport mechanism (Marron Fernandez De Velasco et al., 2015). There are sex differences in the GABA_BR_-GIRK signaling pathway in these neurons. GABA_BR_-dependent GIRK currents in the anterior limbic region of the mPFC were greater in adolescent male mice than in females, but this sex difference was not observed in pyramidal neurons in layer 5/6 of the adjacent limbic cortex.

Previous studies have revealed sex differences in the expression levels of the GABA signaling components, namely, glutamic acid decarboxylase (GAD), GABA receptor subunit, and GABA transporter (GAT) (Pandya et al., 2019). Analysis of sex-specific changes in the expression of GAD, GABA_A/BR_ subunit, and GAT in the human primary sensory and motor cortex revealed sex-dependent differences in the expression of the GABA_AR_ subunit in the superior cerebral gyrus (STG). There is a significant sex-dependent difference in the expression of the α1 subunit of STG: males present significantly higher levels of expression compared to women across all stages of life in STG. Older females had significantly lower α2, α5, and β3 subunit expression in the STG compared to older males. These changes found in the STG may significantly affect GABAergic neurotransmission and lead to sex-specific disease susceptibility and progression (Pandya et al., 2019). There is still a lack of evidence as to whether these baseline differences are involved in the sex-differentiated manifestations of pain and related emotions and behaviors.

In studies on male and female smokers in terms of nicotine dependence, cigarette cravings, and mood or pain sensitivity, researchers used single-photon emission computed tomography (SPECT) to image subjects (Cosgrove et al., 2011). The results showed that females (both female smokers and female non-smokers) had higher GABA_A_-benzodiazepine receptor (GABA_A_-BZR) availability than all males. GABA_A_-BZR availability was negatively correlated with craving and pain sensitivity in female smokers, but not in male smokers. This suggests a sex-specific modulation of GABA_A_-BZR availability and demonstrates the potential of GABA_A_-BZRs to mediate smoking cravings and pain symptoms in female and male smokers (Cosgrove et al., 2011).

It is estimated that GABAergic neurons account for more than half of the hypothalamic neuronal population (Searles et al., 2000), and they may explain some of the structural and functional sex differences observed in the mammalian brain. Studies have reported sex differences in GABA turnover rates in discrete hypothalamic structures in adult rats and determined that these differences may be related to differences in GAD65 and/or GAD67 mRNA levels (Sagrillo and Selmanoff, 1997). There is evidence that GAD65 mRNA levels are significantly higher in female rats in the dorsomedial nucleus (DMN), while GAD67 mRNA levels are higher in male rats in the medial amygdala. These data reveal significant sex differences in GABA turnover and GAD mRNA levels in hypothalamic GABAergic neurons of specific populations (Bowman et al., 2013).

Whether the differential manifestations of these GABAergic neurons participate in the sex differences in pain and pain-related affective behaviors remains to be further studied.

### 3.5. Oxytocin

Oxytocin (Oxt) is a nine-amino-acid peptide hormone that is synthesized and released in the brain primarily by neurons in the paraventricular (PVN) and supraoptic nuclei (SON) of the hypothalamus (Rossoni et al., 2008). Oxt is thought to be associated with pain, and clinically, plasma oxytocin levels are reduced in women with fibromyalgia syndrome (Anderberg and Uvnas-Moberg, 2000). Intranasal administration of Oxt leads to changes in the activity of the bilateral thalamus, left caudate nucleus, and right amygdala, and ameliorates pain in patients with chronic low back pain (Schneider et al., 2020). Epidural oxytocin induces analgesia in patients with severe chronic pain and also improves patients' moods and quality of life.

Oxt has anti-injurious and antinociceptive hormonal effects on neuropathic pain (Xin et al., 2017) induced by nerve injury, which is mediated by its receptor (OTR) and likely occurs due to co-localization of these neurons within OTR-binding sites, such as the spinal dorsal horn (Veronneau-Longueville et al., 1999; Wrobel et al., 2011). Interestingly, in the pain model in rats, Oxt content in the PVN was found to be significantly reduced, but Oxt content in the spinal cord remained unchanged. The researchers also observed that intracerebroventricular injection of Oxt increased the mechanical hypersensitivity response threshold in a dose-dependent manner, whereas intrathecal injection of Oxt did not induce any analgesia. These results indirectly suggest that Oxt in the brain may have an analgesic effect independent of the spinal cord (Zhang et al., 2015). Furthermore, Oxt also plays a crucial role in social behavior, stress, and depression, as verified in animal experiments (Neumann, 2008; Massey et al., 2016). More than one study has suggested that activation of OTR in the VTA is critical for the expression of reward-like properties of social interactions (Song et al., 2016; Borland et al., 2018). However, there are clear sex differences in the embryonic development of Oxt /OTR, which may indicate sex differences in their involvement in pain/behavior (Tamborski et al., 2016; Aulino and Caldwell, 2020). In clinical studies, the expression of Oxt at rs4813625, a single nucleotide polymorphism linked to Oxt was found to correlate more with nociception, anxiety, and wellbeing in females, while no such correlation was found in males (Love et al., 2012).

Another study showed a correlation between depression severity and methylation of the Oxt promoter region. There was a significant negative correlation between critical life events and the mean methylation status as well as the methylation status of single CpG sites in the Oxt promoter region. Whereas, there was no association between depression severity and Oxt methylation. However, there were significant sex differences in the methylation status of Oxt, with females having higher methylation rates than males, suggesting that in patients with depressive disorders, Oxt activation is lower in female patients compared to male ones (Sanwald et al., 2020). Interestingly, in an animal experiment, 3-nitropropionic acid (3-NP)-induced Huntington's disease model was found to have both anxiety and depressive behaviors. 3-NP also reduced the levels of OTR and mGluR2 in the striatum and increased mGluR5. Oxt pretreatment was performed to ameliorate anxiety and depression and to reverse the abnormal expression of OTR, mGluR2, and mGluR5 under the disease state. These behavioral and molecular alterations act similarly between male and female animals (Khodagholi et al., 2022). Meanwhile, some studies support that Oxt may interact with mGluR2 and influence addictive behavior in rats and that this receptor interaction is similar between females and males (Bernheim et al., 2017). However, some researchers have suggested that sex differences in Oxt -regulated emotions tend to occur in the presence of negative stress, for example, one study found that male rats exposed to the stress of social defeat exhibited reduced social avoidance after receiving Intracerebroventricular (ICV) infusions of Oxt (Lukas et al., 2011). Whereas, ICV infusion of Oxt did not reduce social avoidance in stressed female rats (Lukas and Neumann, 2014). The same dose of intranasal Oxt reversed social avoidance in male mice exposed to social defeat, a phenomenon not observed in female mice (Steinman et al., 2016). Nevertheless, sex differences of Oxt in pain affective reactions remain worthy of further study.

## 4. Conclusion

In the study of pain-induced emotional disorders, most animal experiments only use male mice. It is generally considered that many neurological and behavioral functions are affected by estrogen, including emotion, cognitive function, and pain (McEwen and Milner, 2017). Sex hormones, particularly estradiol and progesterone, play an important role in pain perception and mood swings (Vincent and Tracey, 2010). It has been reported that there is a strong link between mood swings and sex hormones, particularly endogenous hormones (Hernandez-Hernandez et al., 2019; Frokjaer, 2020). Menstrual (or estrous) cycles in females altered pain perception (Kaur et al., 2018), depression (Kaur et al., 2018; Zhao et al., 2021), and even neuronal activity in certain brain regions (D'Souza and Sadananda, 2017). Researchers often do not use female mice for research based on these complexities. However, there is also a contrary view. Studies have shown that there is no significant difference in social behavior, depression-like, anxiety-like behavior, and pain threshold in female mice with pain during different estrus periods (Zhao et al., 2021). In any case, it has been reported in the literature that women have higher rates of pain, depression, and anxiety, and the extensive use of male animal experiments has slowed the process of developing drugs that are more suitable for women, such as analgesia, antidepressants, and antianxiety. There is a huge contradiction in this. We cannot ignore these differences. In the future, research needs to include females to further clarify the mechanism of pain-induced emotional disorders and the targets of sex-differentiated regulation of related neurotransmitters and modulation, which is believed to provide better background support for individual precision medicine.

## Author contributions

All authors listed have made a substantial, direct, and intellectual contribution to the work and approved it for publication.
